# Supplementary material for: Explore an open-source value co-creation framework: A multiple case study
Source: PLoS One. 2024 Sep 20;19(9):e0310516. doi: 10.1371/journal.pone.0310516 (PMC11414942; doi:10.1371/journal.pone.0310516)
Supplement: Attachment — Submitted filename: Response to Reviewers.docx [file pone.0310516.s001.docx]

**Response to Reviewers**

Dear Editor and Reviewers,

We sincerely appreciate your valuable time spent providing constructive remarks and useful suggestions, which have significantly enhanced the quality of the manuscript and enabled us to make improvements. Each suggested revision and comment, brought forward by the reviewers was accurately incorporated and considered. Below the comments of the reviewers are responses point by point and the revisions are indicated.

**Reviewer #1:** Some suggestions to the authors:

**Comment 1:** The authors need to highlight in brief why open-source value cocreation(OSVCC) is very important topic in the abstract.

**Reply 1:** We gratefully appreciate your valuable suggestion. We followed your suggestion and added an introduction sentence of OSVCC in the abstract, as follows:

*More and more IT companies and individuals are exploring how to achieve innovation through open-source collaboration, and value co-creation(VCC) in the OSCs has become a trend. Therefore, it is particularly important to examine the mechanism of OSCs under the background of VCC theory. This study proposes a conceptual framework of open-source value co-creation (OSVCC), which is characterized by openness, sharing, collaboration, and freedom, for understanding the internal mechanisms and contextual conditions in the relationship between OSCs participants.*

**Comment 2:** It is not necessary to include the sub-section after the conclusion such as 5.1, 5.2 & 5.3.

**Reply 2:** Thank you for your rigorous advice. We cancelled the sub-sections(5.1, 5.2 & 5.3) as you suggested. Please refer to our manuscript.

**Comment 3:** It is good for the authors to summarize the Conclusion and not too details.

**Reply 3:** We gratefully appreciate your valuable consideration. We remove the detailed description in the conclusion. Please refer to our manuscript.

**Reviewer #2:** The paper describes an Open-Source Value Co-Creation Framework based on case studies. Such an open-source value framework may be invaluable to organizations exploring the pros and cons of adopting open-source software in enterprise systems and the engagement with OS teams.

**Major comments:**

**Comment 1:** Though the case study is confined to a particular geographic location, an attempt should be made to make the findings generalizable. Such an attempt is conspicuously missing in this approach. Please comment on why the authors consider that the framework applies only to one region. More specifically, how is the Chinese context different from the global context?

**Reply 1:** We feel great thanks for your professional review work on our article. According to your nice suggestions, we have made extensive corrections to our previous manuscript. After discussion, we agreed that it is inappropriate to examine the value co-creation mechanism of OSCs in the Chinese context. Since all of OSCs around the world follow the similar open-source rules, with the consent of all authors, we have decided to remove the content in the Chinese context and changed the title of the paper from “Explore an Open-Source Value Co-Creation Framework: A Multiple Case Study in China” to “Explore an Open-Source Value Co-Creation Framework: A Multiple Case Study”. All of these changes are based on the following considerations:

1) TiDB, one of the cases studied in this paper, is an open-source project founded by Chinese individuals. However, more than half of its customers are from outside China and it has a large number of international contributors. Additionally, TiDB is an open-source project incubated by CNCF, a global foundation based in the United States under the Linux Foundation. Therefore, we believe that it is appropriate to examine TiDB in a global context.
2) OpenHarmony, one of the cases studied in this paper, is an open-source project developed by Huawei, a leading global provider of information and communication infrastructure and smart devices. The OpenHarmony-based HarmonyOS has been installed on over 800 million smart devices worldwide and has emerged as the third largest mobile operating system globally, following Android and iOS since 2023. Therefore, it is pertinent to examine OpenHarmony within a global context.
3) The other two cases in this paper involve OpenAnolis, founded by Alibaba, and PaddlePaddle, founded by Baidu. Both companies are listed on the New York Stock Exchange and NASDAQ respectively, representing technology companies from emerging countries in the global capital market. The open-source communities created by these companies have attracted participation from developers worldwide and facilitated communication and cooperation among global developers. Therefore, it is appropriate to consider OpenAnolis and PaddlePaddle within a global context.
4) We believe that the four cases in this study, although all from China, are still representative of the global context. Therefore, we will try to apply the framework of OSVCC to all OSCs.

**Comment 2:** This reviewer considers the lack of interviews as a data collection method, as a major limitation.

**Reply 2:** We sincerely thank you for your valuable feedback that we have used to improve the quality of our manuscript. We discussed your suggestion and invited an open-source expert from Ericsson China R&D Center to join our team. The new author was responsible for developing the interview agenda, inviting interviewees, and participating in the online interview.

The interviews were conducted via video conference during March 2024, and the interview data were used for the theoretical saturation. Using a semi-structured protocol, the interviews were conducted with a total of 14 participants and aimed at collecting the opinions of the respondents on the draft framework. The interviewees came from different organizations with different roles in the OSCs, and each interview involved at least two authors. Each interview lasted between 30 and 40 minutes, and the iFLYREC software was used to convert speech to text. After the interviews, the two authors cross-checked the audio recordings and texts to ensure the accuracy and credibility of the information. The interview information is displayed in the following table.

**Interview information.**

| **OSCs** | **Interviewee** | **Organization** | **Position** | **Duration** |
| --- | --- | --- | --- | --- |
| TiDB | S1  S2  S3 | PingCAP  Dmall  N/A | Marketing Specialist  Database Administrator  Individual Developer | 30 minutes  30 minutes  40 minutes |
| OpenHarmony | S4  S5  S6  S7 | OpenAtom  Huawei  Hoperun  N/A | Operation Consultant  Open-Source Specialist  Software Engineer  Individual Developer | 30 minutes  30 minutes  40 minutes  40 minutes |
| OpenAnolis | S8  S9  S10 | Alibaba  Inspur  Kylin | Open-Source Specialist  Software Engineer  Software Engineer | 30 minutes  40 minutes  40 minutes |
| PaddlePaddle | S11  S12  S13  S14 | Baidu  CMCC  N/A  NA | AI Ecosystem Manager  AI Algorithm Engineer  Individual Developer  Individual Developer | 30 minutes  30 minutes  40 minutes  30 minutes |

Note: All interviewees requested anonymity.

The results of the analysis showed that the concepts and categories obtained have been included in the existing categories, and no new concepts and categories have been generated. Therefore, this paper believes that the conceptual framework of OSVCC has a good theoretical saturation and can reflect the nature of the phenomenon.

**Comment 3:** Theoretical conceptualization to a theory from the described relationship is missing. Perhaps that is the reason for doubts about generalizability.

**Reply 3:** Thank you for your rigorous advice. According to your suggestion, we added a comparative analysis at the end of the case analysis. The specific contents of this part are as follows:

*3.5	Comparative Analysis*

*3.5.1	Comparative Analysis of Maturity*

*Through the comparative analysis of TiDB and OpenHarmony, it is evident that despite both being highly commercialized OSCs, there are notable differences in terms of participant composition and relationships. TiDB has successfully graduated from CNCF, boasting a substantial number of GitHub followers, thus establishing itself as a mature OSC. Conversely, OpenHarmony remains an incubation project under OpenAtom, with community operations overseen by the OpenAtom Foundation. A similar scenario can be observed when comparing OpenAnolis and PaddlePaddle, two low commercialization OSCs. Alibaba intends to donate OpenAnolis to the OpenAtom Foundation for consideration as an open-source incubation project. This highlights the prevalence and significance of open-source foundations as key players during the development stage of OSCs; graduation from such programs serves as a crucial indicator of maturity within these communities.*

*Although OpenAnolis and PaddlePaddle are both low commercialization OSCs, the compositions of participants in these two cases are quite different. OpenAnolis is in the development stage and focuses more on corporate partners in the Operation System field, with fewer individual participants. PaddlePaddle has been established for nearly a decade and has a large number of individual participants, through Github or Gitee to collaborate with contributors. A similar phenomenon is observed in the comparative analysis of TiDB and OpenHarmony. TiDB, in its mature stage, has a large number of individual developers and collaborates with contributors around the world through Github. And TiDB emphasizes the role of TUP, connecting developers and users. The contributors of OpenHarmony are mainly ecological partners around Huawei. Thus, a major difference between OSCs in the mature stage and the development stage is the quantity of individual contributors.*

*3.5.2	Comparative Analysis of Commercialization*

*Comparing OpenHarmony and OpenAnolis, it is evident that despite both being in the development stage, they differ in terms of their commercialization models and participants. The commercialization of OpenHarmony heavily relies on Huawei and its partners, primarily catering to Huawei's "1+8+N" user scenario. On the other hand, OpenAnolis leans more towards public cloud providers for commercialization, with Alibaba being the largest public cloud provider in China. The primary commercial scenario for OpenAnolis revolves around Alibaba's in-house users. This aligns with the findings from a comparative analysis between TiDB and PaddlePaddle at a mature stage. TiDB predominantly achieves commercial success through public cloud services offered to customers within telecommunications, internet, and finance industries. Conversely, PaddlePaddle mainly serves as Baidu's in-house users' main choice for commercial purposes. Henceforth, it can be concluded that public cloud service providers merely serve as channels for OSCs' commercialization rather than major participants themselves. The cases of OpenAnolis and PaddlePaddle demonstrate that founder in-house users are the primary target customers when OSCs have low levels of commercialization.*

**Minor comments**

**Comment 4:** Prior work in the introduction section needs to be organized better, describing how prior work in this domain led the authors to this research question.

**Reply 4:** We gratefully appreciate your valuable suggestion. We have reorganized the Introduction section, added some previous scholars' research, and deleted some confusing descriptions. Here's the latest Introduction, as follows:

*The open-source concept, representing the spirit of new capitalism, is a recent economic phenomenon in information technology(IT) that has emerged in the 21st century (Barron, 2013). The flourishing growth of open-source communities(OSCs) has had a significant impact on the development of the digital economy, such as Linux and Android significantly contribute to the IT infrastructure of the digital economy. Consequently, the value creation of OSCs has attracted the research interest of scholars in the management field. Lin and Maruping (2022) considered the sharing of inward knowledge and the reuse of outward knowledge to be a mechanism for creating value in open-source software. Shaikh and Levina (2019) suggested that the active participation of software users in healthy OSCs can lead to long-term value creation. Mouakhar and Tellier (2017) argued that the open-source software is a global phenomenon that creates value, and it is essential for the world to collaborate in order to protect the values and principles of open-source software.*

*The OSCs are booming, but the principle and mechanism of open-source value creation have not been paid attention to by scholars. The value of open-source is undoubtedly realized through the co-creation of community participants. However, Value Co-Creation(VCC), as a popular theory in the marketing field in recent years, only yielded limited research on OSCs based on this theory. Battistella, Murgia and Nonino (2021)emphasized that open-source value co-creation relies heavily on the contributions of user innovators. Furthermore, the sustainability of corporate participants depends on the reputation of the firm and the improvement of working knowledge. Nagle (2018) elaborates on the idea of gaining competitive advantage by participating in the value co-creation of open-source software. It will not only bring multiple benefits and competitive advantages to individuals or organizations, but also can promote social responsibility and citizenship, bringing more social value to an individual or organization. Based on reviewing nearly two decades of articles on open-source software and value creation, Yang and YS (2023) introduced the concept of open-source value co-creation(OSVCC) which is distinct from customer-dominant logic (Prahalad and Ramaswamy, 2000;Prahalad and Ramaswamy, 2004b) and service-dominant logic (Vargo and Lusch, 2004;Lusch and Vargo, 2006;Vargo and Lusch, 2008). OSVCC is a value co-creation ecosystem characterized by openness, sharing, collaboration, and freedom, which is based on common goals and values, under the rules of OSCs, with open-source code as the carrier and multiple participants collaborative innovation. In OSVCC, open-source participants have the opportunity to actively engage in innovation, designing, programing, as well as decision-making regarding software development. Given that OSVCC serves as a critical foundation for the digital economy, it is imperative to develop a conceptual framework for OSVCC.*

*Scholars have shown sustained interest in researching value co-creation models for the past 20 years, resulting in some research findings. Prahalad and Ramaswamy (2004a) proposed the DART model, which categorized the structure of value co-creation into four subdimensions: dialogue, access, risk, and transparency. After conducting in-depth interviews with executives from 18 large organizations, Payne, Storbacka and Frow (2008) proposed a value co-creation process framework that divided the process into three parts: customer value co-creation, supplier value co-creation, and encounters. Since then, scholars have sequentially refined the framework for the process of value co-creation in knowledge-intensive industries (Aarikka-Stenroos and Jaakkola, 2012) and the mechanism for ecosystems of innovation (Dai, Ye and Dong, 2018). However, the existing value co-creation framework does not target the OSCs. So, it is not suitable for those enterprises and individuals who wish to participate in the OSCs. Therefore, it is important to construct an OSVCC framework, which has significant theoretical and practical implications for understanding the mechanism of value creation within OSCs. The framework could guild programmers who want to participate OSCs, and aid IT enterprises which plan to join OSCs in formulating open-source strategies.*

*In order to address the aforementioned theoretical gap, this study divides the maturity of OSCs into developmental stage and mature stage from the perspective of business ecosystem, and differentiates the commercialization of OSCs into low level and high level from the perspective of open innovation. This study proposes a pairwise combined four-category classification model of OSCs. The model includes four quadrants: high commercial maturity(HCM), high commercial development(HCD), low commercial development (LCD), and low commercial maturity (LCM). Therefore, this paper selects four representative OSCs. Through a comprehensive analysis of each case, this paper summarizes the conceptual framework of OSVCC which building upon the foundation of the classic value co-creation framework and focusing on the participants and their process of value co-creation. The conceptual framework reveals that the crucial factors of OSVCC include three primary participants, three OSVCC processes, three encounter processes, and the principles of OSVCC. The research findings could bridge the theoretical gap and provide valuable insights for understanding the theory of value co-creation in OSCs, and guide enterprises and individuals involved in OSCs.*

**Comment 5:** It is not clear how the two-stage model of OSC relates to business ecosystem theory.

**Reply 5:** We feel great thanks for your professional review work on our article. In order to express the pairwise combined four-category classification model more clearly, we add the content of theoretical analysis before introducing the model. The specific contents are as follows:

*2.1.1	Theoretical Analysis*

*According to the research questions, this paper mainly draws on the business ecosystem theory and open innovation theory to build up a classification model of OCSs. Firstly, the concept of a business ecosystem extends the idea of "ecology" in biology to the field of business. It highlights the symbiotic coexistence of enterprises and stakeholders, as well as the ability of enterprises to lead and cultivate the ecosystem. Moore (1993) initially proposed the concept of the business ecosystem and defined business ecosystem as an "economic union based on organizational interaction". Later, in order to further clarify the internal structural characteristics and evolution mechanism of the business ecosystem, Moore (1998)pointed out that the business ecosystem is a dynamic structural system composed of organizations or groups with certain interests. After that, many scholars have devoted themselves to the study of business ecosystem. Iansiti and Levien (2004)proposed the concept of niche to illustrate the structural characteristics of business ecosystem. From the perspective of enterprise network, Den Hartigh, Tol and Visscher (2006) argued that a business ecosystem is a network of interdependent suppliers and customers around a certain core technology. Senyo, Liu and Effah (2019)introduced that the digital business ecosystem is an extension of the business ecosystem, and the core enterprises could create value by building the digital business ecosystem. OSCs are typical digital business ecosystem, which are online communities around core technology and follow the law of business ecosystem. Therefore, it is appropriate to analyze OSCs from the perspective of business ecosystem theory.*

*Secondly, for a long time, enterprises have firmly believed that internal research and development capabilities are valuable strategic assets, emphasizing that successful innovation requires strong control. However, Chesbrough (2003)held a different view and proposed the theory of open innovation through research on high tech companies, advocating for companies to intentionally harness both internal and external innovation. Baldwin and Von Hippel (2011)explored the paradigm shift in innovation patterns by comparing three innovation paradigms: single user or company innovation, open collaborative innovation, and producer innovation, emphasizing the importance of new business models that support user innovation and open collaborative innovation. Subsequently, Chesbrough and Bogers (2014)expanded the concept of open innovation and proposed that open innovation can be realized through different types of profit and non-profit mechanisms, which is a distributed innovation process that crosses organizational boundaries and effectively manages knowledge flows. OSCs are distributed innovation paradigms formed through open-source collaboration, and OSCs’ participants achieve innovation through knowledge flow across organizational boundaries. Therefore, open-source communities can be analyzed in the context of open innovation theory.*

*In a word, business ecosystem theory and open innovation theory can be well applied to the research of OSCs. Business ecosystem theory provides a dynamic perspective that helps enterprises position themselves in an open-source environment, as well as establish collaborative relationships with other members of the ecosystem. Open innovation theory encourages IT enterprises to accelerate innovation processes through sharing of code, knowledge, and best practices. By participating in OSCs, enterprises could absorb external innovation resources, and at the same time enhance their influence and competitiveness in the commercial market by contributing their own innovation achievements.*

**Comment 6:** The "commercial dimension" in HCM seems to be ill-defined.

**Reply:** We gratefully appreciate your careful review. We found the previous description to be somewhat confusing, so we have removed it and replaced it with the following:

*In the developmental phase, OSCs have constructed a collaborative ecosystem and continue to attract a variety of participants. In the mature phase, OSCs have established stable cooperation among participants, gained influence in specific areas, and even become leaders in segment technology areas. The degree of commercialization is usually determined by the founder of the OSC. Founders who choose low commercialization often have alternative source of revenue and strong financial resources that prioritize the long-term benefits of their open-source strategy. On the other hand, founders who choose to be highly commercial often focus solely on open-source projects or are enticed by significant market profits from open-source commercialization.*

**Reviewer #3:** It’s a pleasure to review this manuscript addressing a significant issue. Some suggestions are proposed as follows.

**Comment 1:** Since value co-creation is the essential concept of this study, it is expected to have more support from the literature.

**Reply:** We gratefully appreciate your valuable suggestion. Since there are few studies on the value co-creation of OSCs, we only review two recent articles on the value co-creation of OSCs, which are as follows:

*However, Value Co-Creation(VCC), as a popular theory in the marketing field in recent years, only yielded limited research on OSCs based on this theory. Battistella, Murgia and Nonino (2021)emphasized that open-source value co-creation relies heavily on the contributions of user innovators. Furthermore, the sustainability of corporate participants depends on the reputation of the firm and the improvement of working knowledge. Nagle (2018) elaborates on the idea of gaining competitive advantage by participating in the value co-creation of open-source software. It will not only bring multiple benefits and competitive advantages to individuals or organizations, but also can promote social responsibility and citizenship, bringing more social value to an individual or organization.*

**Comment 2:** The authors proposed an excellent classification model based on the business ecosystem and open innovation theory; readers would expect some discussion on the rationale and background.

**Reply:** We feel great thanks for your professional review work on our article. To more clearly describe the theoretical basis, we add a theoretical analysis before introducing the classification model, the specific content is as follows:

*2.1.1	Theoretical Analysis*

*According to the research questions, this paper mainly draws on the business ecosystem theory and open innovation theory to build up a classification model of OCSs. Firstly, the concept of a business ecosystem extends the idea of "ecology" in biology to the field of business. It highlights the symbiotic coexistence of enterprises and stakeholders, as well as the ability of enterprises to lead and cultivate the ecosystem. Moore (1993) initially proposed the concept of the business ecosystem and defined business ecosystem as an "economic union based on organizational interaction". Later, in order to further clarify the internal structural characteristics and evolution mechanism of the business ecosystem, Moore (1998)pointed out that the business ecosystem is a dynamic structural system composed of organizations or groups with certain interests. After that, many scholars have devoted themselves to the study of business ecosystem. Iansiti and Levien (2004)proposed the concept of niche to illustrate the structural characteristics of business ecosystem. From the perspective of enterprise network, Den Hartigh, Tol and Visscher (2006) argued that a business ecosystem is a network of interdependent suppliers and customers around a certain core technology. Senyo, Liu and Effah (2019)introduced that the digital business ecosystem is an extension of the business ecosystem, and the core enterprises could create value by building the digital business ecosystem. OSCs are typical digital business ecosystem, which are online communities around core technology and follow the law of business ecosystem. Therefore, it is appropriate to analyze OSCs from the perspective of business ecosystem theory.*

*Secondly, for a long time, enterprises have firmly believed that internal research and development capabilities are valuable strategic assets, emphasizing that successful innovation requires strong control. However, Chesbrough (2003)held a different view and proposed the theory of open innovation through research on high tech companies, advocating for companies to intentionally harness both internal and external innovation. Baldwin and Von Hippel (2011)explored the paradigm shift in innovation patterns by comparing three innovation paradigms: single user or company innovation, open collaborative innovation, and producer innovation, emphasizing the importance of new business models that support user innovation and open collaborative innovation. Subsequently, Chesbrough and Bogers (2014)expanded the concept of open innovation and proposed that open innovation can be realized through different types of profit and non-profit mechanisms, which is a distributed innovation process that crosses organizational boundaries and effectively manages knowledge flows. OSCs are distributed innovation paradigms formed through open-source collaboration, and OSCs’ participants achieve innovation through knowledge flow across organizational boundaries. Therefore, open-source communities can be analyzed in the context of open innovation theory.*

*In a word, business ecosystem theory and open innovation theory can be well applied to the research of OSCs. Business ecosystem theory provides a dynamic perspective that helps enterprises position themselves in an open-source environment, as well as establish collaborative relationships with other members of the ecosystem. Open innovation theory encourages IT enterprises to accelerate innovation processes through sharing of code, knowledge, and best practices. By participating in OSCs, enterprises could absorb external innovation resources, and at the same time enhance their influence and competitiveness in the commercial market by contributing their own innovation achievements.*

**Comment 3:** The quality aspect of the case study methodology could be addressed.

**Reply:** We gratefully appreciate your valuable suggestion. In order to improve the quality of the manuscript, we added interviews with participants in the OSCs as a source of data to test theoretical saturation. We invited an open-source expert from Ericsson China R&D Center to join our team. The new author was responsible for developing the interview agenda, inviting interviewees, and participating in the online interview.

The interviews were conducted via video conference during March 2024, and the interview data were used for the theoretical saturation. Using a semi-structured protocol, the interviews were conducted with a total of 14 participants and aimed at collecting the opinions of the respondents on the draft framework. The interviewees came from different organizations with different roles in the OSCs, and each interview involved at least two authors. Each interview lasted between 30 and 40 minutes, and the iFLYREC software was used to convert speech to text. After the interviews, the two authors cross-checked the audio recordings and texts to ensure the accuracy and credibility of the information. The interview information is displayed in the following table.

**Interview information.**

| **OSCs** | **Interviewee** | **Organization** | **Position** | **Duration** |
| --- | --- | --- | --- | --- |
| TiDB | S1  S2  S3 | PingCAP  Dmall  N/A | Marketing Specialist  Database Administrator  Individual Developer | 30 minutes  30 minutes  40 minutes |
| OpenHarmony | S4  S5  S6  S7 | OpenAtom  Huawei  Hoperun  N/A | Operation Consultant  Open-Source Specialist  Software Engineer  Individual Developer | 30 minutes  30 minutes  40 minutes  40 minutes |
| OpenAnolis | S8  S9  S10 | Alibaba  Inspur  Kylin | Open-Source Specialist  Software Engineer  Software Engineer | 30 minutes  40 minutes  40 minutes |
| PaddlePaddle | S11  S12  S13  S14 | Baidu  CMCC  N/A  NA | AI Ecosystem Manager  AI Algorithm Engineer  Individual Developer  Individual Developer | 30 minutes  30 minutes  40 minutes  30 minutes |

Note: All interviewees requested anonymity.

The results of the analysis showed that the concepts and categories obtained have been included in the existing categories, and no new concepts and categories have been generated. Therefore, this paper believes that the conceptual framework of OSVCC has a good theoretical saturation and can reflect the nature of the phenomenon.

**Comment 4:** Readers would like more explanations on the proposed conceptual framework of OSVCC.

**Reply:** Thank you for your rigorous advice. We have modified the description of the framework by adding some descriptions, please refer to below:

*In this framework, the participants in the OSVCC are more specific and explicit compared with the classical value co-creation framework. The DP typically initiates open-source projects, but the DP neither owns the final product nor has an employer-employee relationship with the other participants (Snarby and Abrahamsson, 2017). DP serves as the primary driving force behind the OSCs’ development and reaps the most significant social and commercial benefits. EPs have a complementary role in the open-source community, and their relationship with the DP is delicate (Lan, Liu and Dong, 2019). EPs primarily comprise the upstream and downstream enterprises of the DP's value chain. IPs frequently serve as the most active participants in the framework, deriving great satisfaction from contributions (Von Krogh et al., 2012). IPs join the community on the basis of their interests or career development. These three primary participants and the interactions between them constitute the crucial factors of OSVCC. Participants collaborate and work interdependently, while maintaining their independence to create value, guided by the principles of openness, sharing, collaboration, and freedom.*

**Comment 5:** The managerial implications could discuss the implementation issues regarding the value co-creation of OSCs.

**Reply:** We gratefully appreciate your valuable suggestion. We have modified the content of managerial implications by mainly adding a fourth management recommendation on open-source implementation. Please refer to the following content:

*The conceptual framework of OSVCC have four managerial implications for enterprise. Firstly, the complexity of the OSVCC process challenges traditional value co-creation management practices. The relationship between the participants in value co-creation is no longer the supplier-customer relationship, but the triangular relationship. The enterprise's decision may affect the other two participants and ultimately affect the stability of the triangular relationship. Secondly, the framework shows that the enterprises choose different focuses in OSVCC. If an enterprise is the founder of the OSC, the enterprise should focus on capturing opportunities, formulating plans, implementing and evaluating. If an enterprise is an OSC user or developer, it should focus on following the domination enterprise and looking for opportunities and then taking actions based on its own cognitive. Thirdly, this research stresses the importance of organization learning. No matter what kind of role, an enterprise needs to break through organizational boundaries to learn and exchange knowledge when participating in OSVCC. Finally, the implementation issues of the OSVCC should be of concern. The participants need to understand the process of OSVCC and take the initiative to design and deliver relevant relational experiences. Our research also stresses the importance of organizational learning and knowledge management by managing and designing the encounter process from a long-term perspective.*

**Comment 6:** Authors comment on one of the limitations of their study, which only examined four Chinese OSCs, which might differ from other well-known open-source projects born in the United States. After this study, it seems they should have some standpoints.

**Reply:** We feel great thanks for your professional review work on our article. According to your nice suggestions, we have made extensive corrections to our previous manuscript. After discussion, we agreed that it is inappropriate to examine the value co-creation mechanism of OSCs in the Chinese context. Since all of OSCs around the world follow the similar open-source rules, with the consent of all authors, we have decided to remove the content in the Chinese context and changed the title of the paper from “Explore an Open-Source Value Co-Creation Framework: A Multiple Case Study in China” to “Explore an Open-Source Value Co-Creation Framework: A Multiple Case Study”. All of these changes are based on the following considerations:

1) TiDB, one of the cases studied in this paper, is an open-source project founded by Chinese individuals. However, more than half of its customers are from outside China and it has a large number of international contributors. Additionally, TiDB is an open-source project incubated by CNCF, a global foundation based in the United States under the Linux Foundation. Therefore, we believe that it is appropriate to examine TiDB in a global context.
2) OpenHarmony, one of the cases studied in this paper, is an open-source project developed by Huawei, a leading global provider of information and communication infrastructure and smart devices. The OpenHarmony-based HarmonyOS has been installed on over 800 million smart devices worldwide and has emerged as the third largest mobile operating system globally, following Android and iOS since 2023. Therefore, it is pertinent to examine OpenHarmony within a global context.
3) The other two cases in this paper involve OpenAnolis, founded by Alibaba, and PaddlePaddle, founded by Baidu. Both companies are listed on the New York Stock Exchange and NASDAQ respectively, representing technology companies from emerging countries in the global capital market. The open-source communities created by these companies have attracted participation from developers worldwide and facilitated communication and cooperation among global developers. Therefore, it is appropriate to consider OpenAnolis and PaddlePaddle within a global context.

We believe that the four cases in this study, although all from China, are still representative of the global context. Therefore, we will try to apply the framework of OSVCC to all OSCs.
