# Supplementary material for: Explore an open-source value co-creation framework: A multiple case study
Source: PLoS One. 2024 Sep 20;19(9):e0310516. doi: 10.1371/journal.pone.0310516 (PMC11414942; doi:10.1371/journal.pone.0310516)
Supplement: Attachment — Submitted filename: Response to Reviewers.docx [file pone.0310516.s002.docx]

**Responds to Reviewers**

Dear Editor and Reviewers,

We sincerely appreciate your valuable time spent providing constructive remarks and useful suggestions, which have significantly enhanced the quality of the manuscript and enabled us to make improvements. Each suggested revision and comment, brought forward by the reviewers was accurately incorporated and considered. Below the comments of the reviewers are responses point by point and the revisions are indicated.

Sincerely

Yang Luo

**Reviewer #1:**

**Comment:** To improve some of the citations especially for the article having more than 2 authors. For example Payne, Storbacka and Frow (2008) should be replaced with Payne et al. (2008)

**Response:** We sincerely thank you for your valuable feedback that we have used to improve the quality of our manuscript. According to your comment, we changed the citations format to limit within two authors showing in the paper.

**Reviewer #3:** It’s a great pleasure to review this manuscript, which addresses issues I am interested in. My primary concerns are as follows.

**Comment 1:** As value co-creation (VCC) is the core concept of this study, a solid theoretical background could be supported first. Then come the features and challenges of open-source communities that lead to the introduction of the OSVCC.

**Response 1:** Thank you for your rigorous advice, we have revised the article. First of all, we modify the second paragraph of the introduction section, add the difficulties encountered in the application of value co-creation theory in the open-source community, and introduce the concept of OSVCC. Then, we add the section 2 Theoretical Underpinnings, and focus on the research perspective of value co-creation theory in 2.1, as well as why the current research framework of value co-creation could not be applied to the study of open-source community. Please refer to the following section for specific modifications.

1）The second paragraph of the introduction section

*The value of open-source is undoubtedly realized through the co-creation of community participants. Value co-creation(VCC) theory is a consumer-centered theory in which suppliers and consumers co-create value[5]. It emphasizes that value is co-created through the interaction and cooperation among suppliers, consumers, partners, as well as other stakeholders[6]. However, we find that OSCs are rarely examined under the framework of VCC. There is no supplier-customer relationship in the OSCs, which may not apply VCC theory. OSCs are the unique phenomenon to the IT industry, and the existing VCC framework could not cover such segmented areas. In order to address the above problem of adaptation, Yang and YS [7] introduced the concept of Open-Source Value Co-Creation(OSVCC) which is a VCC system based on common goals and values and following the rules of OSCs, and emphasized that OSVCC is distinct from customer experience[8] and service-dominant(S-D) logic[9]. OSVCC, as a "beyond software" paradigm, prompts the IT companies to shift from product or service focus to engagement focus[10]. Given that OSVCC serves as a critical foundation for the digital economy, it is imperative to develop a conceptual framework for OSVCC.*

2）2.1 Value Co-Creation in Open-Source Community

*After more around 20 years of development, VCC theory has formed two main research perspectives[11]. One research perspective of the VCC theory is based on customer experience, and it mainly focuses on the creation of personalized customer experience value through the interaction between customers and suppliers[8,12]. However, there are only participants and contributors, and no clear customer-supplier distinction in the OSCs. Another one is based on S-D logic[9,13], including service logic, service science, and service ecosystem[14]. The S-D logic emphasizes that service exchange is the core of value creation, and value is determined and co-created by customers[15,16]. The S-D logic is an economic model proposed based on the macro perspective of economic development and evolution mode[17] and rarely guides individuals and enterprises from the micro perspective of enterprise strategic management[18].*

*Therefore, it is difficult for the two mainstream research perspectives of VCC to be directly applied to the research of OSCs, but a few scholars have made some attempts. Battistella, et al. [19]emphasized that VCC in OSCs relies heavily on the contributions of user innovators, and the sustainability of enterprise participants depends on the reputation of the firm and the improvement of working knowledge. Nagle [20] elaborates on the idea of gaining a competitive advantage by participating in the VCC of open-source software. It will not only bring multiple benefits and competitive advantages to individuals or organizations but also can promote social responsibility and citizenship, bringing more social value to an individual or organization [20,21]. Based on reviewing nearly two decades of articles on open-source software and value creation, Yang and YS [7] proposed OSVCC which is a VCC ecosystem characterized by openness, sharing, collaboration, and freedom, with open-source code as the carrier and multiple participants collaborative innovation. In OSVCC, open-source participants have the opportunity to actively engage in innovation, designing, programming, as well as decision-making regarding software development[7]. Compared to customer experience and S-D logic, OSVCC is more specific, focuses on VCC in OSCs, and emphasizes contributors co-create value through collaboration in OSCs(see Table 1).*

**Table 1.** Comparison of the VCC research perspective.

|  | **Customer experience** | **S-D logic** | **OSVCC** |
| --- | --- | --- | --- |
| **Perspective** | Enterprises’ strategy and competition | Economic development and evolution | Open-source community |
| **Scope** | Intermediate | Marco | Micro |
| **Value base** | Personalized experience | Service-use | Collaboration |
| **Co-creators** | Customers and suppliers | Customers and suppliers | Open-Source participants |
| **Initiative** | Customers | Suppliers | Everyone |

Note: The authors compiled this table.

**Comment 2:** Since VCC is an essential part of service-dominant logic, the authors could comment on the differences between service-dominant, customer-dominant, and OSVCC.

**Response 2:** We gratefully appreciate your valuable consideration. We added the section 2 Theoretical Underpinnings, which includes a comparison of customer-dominant logic, service-dominant logic and open source value co-creation(OSVCC). Please refer to following content:

*After more around 20 years of development, VCC theory has formed two main research perspectives[11]. One research perspective of the VCC theory is based on customer experience, and it mainly focuses on the creation of personalized customer experience value through the interaction between customers and suppliers[8,12]. However, there are only participants and contributors, and no clear customer-supplier distinction in the OSCs. Another one is based on S-D logic[9,13], including service logic, service science, and service ecosystem[14]. The S-D logic emphasizes that service exchange is the core of value creation, and value is determined and co-created by customers[15,16]. The S-D logic is an economic model proposed based on the macro perspective of economic development and evolution mode[17] and rarely guides individuals and enterprises from the micro perspective of enterprise strategic management[18].*

*Therefore, it is difficult for the two mainstream research perspectives of VCC to be directly applied to the research of OSCs, but a few scholars have made some attempts. Battistella, et al. [19]emphasized that VCC in OSCs relies heavily on the contributions of user innovators, and the sustainability of enterprise participants depends on the reputation of the firm and the improvement of working knowledge. Nagle [20] elaborates on the idea of gaining a competitive advantage by participating in the VCC of open-source software. It will not only bring multiple benefits and competitive advantages to individuals or organizations but also can promote social responsibility and citizenship, bringing more social value to an individual or organization [20,21]. Based on reviewing nearly two decades of articles on open-source software and value creation, Yang and YS [7] proposed OSVCC which is a VCC ecosystem characterized by openness, sharing, collaboration, and freedom, with open-source code as the carrier and multiple participants collaborative innovation. In OSVCC, open-source participants have the opportunity to actively engage in innovation, designing, programming, as well as decision-making regarding software development[7]. Compared to customer experience and S-D logic, OSVCC is more specific, focuses on VCC in OSCs, and emphasizes contributors co-create value through collaboration in OSCs(see Table 1).*

**Table 1.** Comparison of the VCC research perspective.

|  | **Customer experience** | **S-D logic** | **OSVCC** |
| --- | --- | --- | --- |
| **Perspective** | Enterprises’ strategy and competition | Economic development and evolution | Open-source community |
| **Scope** | Intermediate | Marco | Micro |
| **Value base** | Personalized experience | Service-use | Collaboration |
| **Co-creators** | Customers and suppliers | Customers and suppliers | Open-Source participants |
| **Initiative** | Customers | Suppliers | Everyone |

Note: The authors compiled this table.

**Comment 3:** VCC models could be presented in a more organized manner, stressing this study's differentiated advantages.

**Response 3:** We feel great thanks for your professional review work on our article. At the end of the introduction section, we analyzed the existing value co-creation models in a more organized manner to highlight the differentiated advantages of this study.

*To bridge the aforementioned theoretical gap, we begin with a pairwise combined four-category classification model along the dimensions of maturity and commercialization. Building on this model, we select four typical cases and detail our case analysis, and then propose a conceptual framework of OSVCC. We discover the relationship between the participants of OSVCC is no longer a supplier-customer relationship, but a triangle relationship, and the decision of either party may affect the stability of the triangle relationship. This framework stresses the importance of open-source collaboration and organization learning in OSCs. Our findings bridge the theoretical gap, provide valuable insights for understanding the VCC theory in OSCs, and guide enterprises and individuals involved in OSCs. Finally, we discuss both theoretical and managerial implications.*

**Comment 4:** The authors could defend their combining use of the business ecosystem theory and the open innovation theory with in-depth elaborations.

**Response 4:** We gratefully appreciate your valuable suggestion. We explained in depth why we combined business ecosystem theory and open innovation theory to construct the classification model in 3.1	Classification Model. Please refer to the following content for specific modification

*According to the research questions, this paper mainly draws on the business ecosystem theory and open innovation theory to build up a classification model of OSCs. This classification model is to clarify the situational differences and conditions of OSCs, divides all OSCs into four categories, and provides a theoretical basis for case analysis.*

*Moore [24] introduced the concept of the business ecosystem and categorized its development into four stages: birth, expansion, leadership, and self-renewal. OSCs are ecosystems in which multiple participants collaborate to create and share value through innovative collaboration. This ecosystem also experiences a gradual process of development and maturity. Based on the research of open source software development process, Petrinja, et al. [32] proposed the open-source maturity model (OMM) and divided it into three maturity levels: basic, intermediate, and advanced. Kuwata, et al. [33] also categorized the development of OSCs into five stages: initial, managed, defined, quantitatively managed, and optimized. As technology advances and market needs change, the participants in OSCs continuously adapt their strategies to the changing ecosystem environment. In the early stages, the participants could focus more on the sharing of technology and the rapid iteration of code[34]. As ecosystems mature, they could shift to a greater focus on collaboration and standardization to ensure compatibility and interoperability between different components. With the increasing influence, participants could focus more on business model innovation and explore how to make money by providing value-added services, customizing solutions, or building specific platforms[19]. At the same time, they will pay close attention to user feedback and market trends, so as to timely adjust products and services to meet user needs. Ultimately, as the ecosystem stabilizes, participants are likely to place more emphasis on maintaining and optimizing the existing system, while continuing to explore new technological areas to keep the ecosystem dynamic and competitive[2]. This study introduces a two-stage model for OSCs: the developmental stage and the mature stage. The developmental stage aligns with Moore [24] proposed concept of birth and expansion, while the mature stage requires stability and the production of reliable open-source software products.*

*West and Lakhani [35] were the first to introduce the idea that the research paradigm of open innovation applies not only to enterprises but also to the study of OSCs. Morgan and Finnegan [36] argued that open-source software represents the most developed form of open innovation, and highlighted the benefits of engaging in OSCs, such as reducing costs through inward participation and creating value through outward participation. Chesbrough [37] believes that to obtain innovation profits, enterprises not only need technological innovation but also need appropriate business models to commercialize the technology. Enterprises can generate value by commercializing technology, and make strategic choices regarding open-source technologies [38]. Chesbrough and Schwartz [39]highlights the importance of co-development partnerships in business models, which are mutually beneficial working relationships between two or more parties aimed at creating and providing new products, technologies, or services. This partnership could allow OSCs to be commercialized without waiting for the technology to mature. The enterprise that dominates the community influences the commercialization of the community, leading to varying degrees of commercialization. When the dominant enterprise chooses to start commercialization, it usually needs to consider its own business situation[40] and the co-development relationships between participants in the community[41]. Therefore, we classify OSCs into two grades in the commercialization dimension: low and high.*

*According to the above analysis, OSCs can be classified into two dimensions: maturity and commercialization. In the developmental phase, OSCs have constructed a collaborative ecosystem and continue to attract a variety of participants. In the mature phase, OSCs have established stable cooperation among participants, gained influence in specific areas, and even become leaders in segment technology areas. The degree of commercialization is usually determined by the founder of the OSC. Founders who choose low commercialization often have alternative sources of revenue and strong financial resources that prioritize the long-term benefits of their open-source strategy. On the other hand, founders who choose to be highly commercial often focus solely on open-source projects or are enticed by significant market profits from open-source commercialization. We divide all OSCs into four categories through the two dimensions of maturity and commercialization. The resulting classification model for the OSCs is presented in Figure 1.*

**Figure 1.** The classification model of OSCs.

**Comment 5:** The case selection criteria might be vague and ambiguous; therefore, some concrete requirements are suggested.

**Response 5:** We gratefully appreciate your careful review. We added several objective indicators for case selection.

*This study adopted the theoretical sampling method for case selection, which involved selecting and collecting the raw data according to the research objectives. This study primarily investigated the operational mechanism through the dimensions of maturity and commercialization of OSCs to develop a conceptual framework. Four OSCs were selected on the basis of the research questions and analytical method, using the following screening criteria:*

*1) The selected cases must be OSCs that have been continuously operating since their establishment and have organized offline community activities at least once every six months;*

*2)	The selected cases must be typical and representative OSCs from different domains with global influence, organizing or participating in at least one global technology workshop or summit in this domain every year;*

*3)	The selected cases must have been established for more than two years and have sufficient online information for research.*

*Based on the above criteria, the four OSCs selected were TiDB, OpenHarmony, OpenAnolis, and PaddlePaddle. Table 2 presents the background information for each project.*

**Table 2.** The background information for the selected OSCs.

|  | **TiDB** | **OpenHarmony** | **OpenAnolis** | **PaddlePaddle** |
| --- | --- | --- | --- | --- |
| **Setup** | 2015 | 2020 | 2021 | 2016 |
| **Founder** | PingCAP | Huawei | Alibaba | Baidu |
| **Domain** | Database | Mobile OS | Server OS | AI |
| **Headquarters** | Beijing | Shenzhen | Hangzhou | Beijing |
| **Employees** | <300 | ≈200,000 | ≈240,000 | ≈40,000 |
| **Foundation** | CNCF | OpenAtom | None | None |

Note: The authors compiled this table using publicly available information.

**Comment 6:** The comparative analyses could be conducted more formally and systematically.

**Response 6:** Thank you for your rigorous advice. We revised the comparative analysis section and added 4.5 Comparative Analysis of Primary Participants. We first identified the primary player in OSVCC, then compared and analyzed the imbalance distribution of the number of the three types of primary participants in each case, and analyzed the reasons for this imbalance.

*4.5	Comparative Analysis of Primary Participants*

*The realization of VCC varies depending on the differences in the participant's relationships. Hence, it is essential to consider the variations between the participants. According to the analysis of the aforementioned four cases, this study posits that the following three roles are the primary participants in OSCs:*

*1)	The dominant participant(DP), which is usually the initiator, exerts influence over the development of the OSCs. The DP plays a pivotal role in driving OSCs, reaping significant social and commercial benefits [46];*

*2)	Enterprise participants (EPs) consist of developers and users from enterprises in the OSCs. Enterprise developers (EDs) primarily consist of upstream and downstream companies in the supply chain of the DP. Enterprise users (EUs) typically prioritize the performance and usability of open-source products [46], and EUs with development capabilities are also EDs;*

*3)	Individual participants (IPs) consist of independent developers and users who are not affiliated with corporations. Generally, individual developers(IDs) also serve as individual users (IUs), whose engagement in OSCs is motivated by personal interests or career development [46-48].*

*In the TiDB case, the DP, PingCAP, has profited from a high degree of commercialization, so it continues to provide sufficient support for the community and actively participates in OSVCC. Mature communities could provide rapid response and powerful supporting facilities for EUs, so EUs actively participate in OSVCC; however, higher maturity suppresses the space in which enterprises can participate and inhibits their willingness to develop, so the number of EDs in the community is small. IUs could get more effective support in mature communities, so they are willing to actively participate in contributions; at the same time, the participation of IDs in well-known open-source projects brings them prestige and job opportunities, so IPs actively participate in OSVCC.*

*In the OpenHarmony case, although the DP, Huawei, has benefited from commercialization, the community is still in the developmental stage and needs to attract more participants to make positive contributions. The community is not mature yet, but it has certain commercialization prospects, which means market opportunities, so EDs and EUs actively participate in OSVCC. Higher commercialization prospects could attract IDs, but the community is still in the developmental stage and the supporting facilities are not ready, resulting in fewer IDs and IUs joining in OSVCC.*

*In the case of OpenAnolis, the open-source software has already been used internally for several years, and the DP, Alibaba, could contribute accumulated experience to the community to accelerate OSVCC collaboration. Since the community is still in its infancy and the commercial prospects are unclear, EUs are usually not actively involved in OSVCC. However, considering the huge internal use demand of DP, EDs join in OSVCC collaboration under the influence of DP. In this case, IDs and IUs are mainly participating in the community out of interest, and a small quantity.*

*In the case of PaddlePaddle, the software has widely and fully utilized the DP’s internal and external resources, but there are shortcomings in commercial promotion. EDs prefer to use the mainstream AI framework in large-scale commercial use scenarios and try to participate in OSVCC in small-scale scenarios. Due to personal interest, the popularity of the community, and the complete supporting facilities, IPs and IUs actively participate in OSVCC collaboration.*

**Ta****ble 5.** Participants in OSCs.

| **Participants** | | **HCM**  **(TiDB)** | **HCD**  **(OpenHarmony)** | **LCD**  **(OpenAnolis)** | **LCM**  **(PaddlePaddle)** |
| --- | --- | --- | --- | --- | --- |
| **DP** | | PingCAP | Huawei | Alibaba | Baidu |
| **EPs** | **EDs** | Few | Hoperun, Pateo | Intel, Uniontech | Few |
|  | **EUs** | Meituan, Zhihu | Huawei, Midea | Alibaba | Baidu, JD |
| **IPs** | **IDs** | Numerous | Few | Few | Numerous |
|  | **IUs** | Numerous | Few | Few | Numerous |

Note: The authors compiled the table using the information from the official website of the OSCs.

**Comment 7:** The conclusions could stress the theoretical and managerial implications more significantly.

**Response 7:** We sincerely thank you for your valuable feedback that we have used to improve the quality of our manuscript. We revised the conclusion section of the article to better highlight the theoretical contributions and managerial implications of the article.

*The open-source concept has emerged as an important software development model in the IT industry, and the VCC has been widely embraced as a marketing strategy. However, there is a scarcity of research regarding VCC by OSCs. From a theoretical perspective, this research fills the gap in the existing literature. On the one hand, we systematically constructed the research framework of OSVCC and found the triangular relationship. This framework inherits the widely adopted VCC process model and emphasizes a view of the collaboration of the participants. On the other hand, we creatively combined the business ecosystem theory and the open innovation theory to build a four-quadrant classification model of OSCs. This classification model enables us to examine the existing OSCs from two dimensions: maturity and commercialization.*

*From a practical perspective, this research provides four managerial implications for the enterprise. Firstly, the complexity of the OSVCC process challenges traditional VCC management practices. The relationship between the participants in VCC is no longer the supplier-customer relationship, but the triangular relationship. The enterprise's decision may affect the other two participants and ultimately affect the stability of the triangular relationship. Secondly, the framework shows that the enterprises choose different focuses in OSVCC. If an enterprise is the founder of the OSC, the enterprise should focus on capturing opportunities, formulating plans, implementing, and evaluating. If an enterprise is an OSC user or developer, it should focus on following the domination enterprise, looking for opportunities and taking actions based on its own cognition. Thirdly, this research stresses the importance of organizational learning. No matter what kind of role, an enterprise needs to break through organizational boundaries to learn and exchange knowledge when participating in OSVCC. Finally, the implementation issues of the OSVCC should be of concern. The participants need to understand the process of OSVCC and take the initiative to design and deliver relevant relational experiences. Our research also stresses the importance of organizational learning and knowledge management by managing and designing the encounter process from a long-term perspective.*
